# Factors associated with therapy non-completion for children with problematic sexual behaviors

**DOI:** 10.3389/frcha.2024.1322578

**Published:** 2024-10-09

**Authors:** Camille Pitre, Isabelle Daignault, Stéphanie Chouinard Thivierge, Marc Tourigny

**Affiliations:** ^1^ School of Criminology, Montreal University, Montreal, QC, Canada; ^2^ School of Social Work and Criminology, Université Laval, Québec, QC, Canada; ^3^ Psychoeducation Department, Sherbrooke University, Longueuil, QC, Canada

**Keywords:** problematic sexual behaviors, children, cognitive behavioral therapy, therapy non-completion, family

## Abstract

**Introduction:**

Children with problematic sexual behaviors (PSBs) can benefit, along with their parents or caregivers, from specialized therapeutic services to limit the manifestation of these behaviors. However, for some families, mobilization for therapy represents a significant challenge since a considerable proportion do not complete the therapy intended for them. The present study aims to identify the factors associated with therapy completion, thus allowing a deeper understanding of how to support children and more broadly families to complete their therapeutic process.

**Methodology:**

The sample consists of 67 caregiver-child dyads referred to a specialized Center offering PSBs-focused cognitive behavioral therapy, actively involving the caregiver. Standardized questionnaires were administered to children and their caregiver before and after the therapy.

**Results:**

While non-completers represent 31% of our sample, they present very similar profiles to completers in terms of socio-demographic characteristics, behavior problems and symptoms. However, they appear to differ regarding living situations and coping mechanisms. Results show that children who complete therapy are more likely to live in a placement situation, compared to non-completers. Children who completed therapy also report using more coping strategies that aim toward getting social support and less distancing coping strategies than non-completers. Non completers also reported feeling less maternal support compared to completers.

**Discussion:**

Results underline the importance of implementing mobilization efforts for families with children with PSBs, along with a focus on developing efficient coping mechanisms.

## 1 Introduction

The emergence of sexuality in some children can be worrying. This is the case when they present problematic sexual behaviors (PSBs) towards other children and when these behaviors are characterized by signs indicative of severity such as coercion or persistence despite interventions, or when the behaviors cause emotional distress, in the child manifesting the behavior or in the victim (1). As defined by the ATSA task force (Association for Treatment and Prevention of Sexual Abuse), PSBs refer to “children aged 12 and younger who initiate behaviors involving sexual body parts (i.e., genital, anus, buttocks, or breasts) that are developmentally inappropriate or potentially harmful to themselves or others” (2). As recommended by this task force, when children exhibit PSBs, rapid management with active parental involvement is recommended. However, a high non-completion rate is observed in most studies that have assessed the effects of these specialized interventions (3–6). Because not completing therapy often stem from family factors that are external to the child, and that several family issues can influence the decision of not completing therapy, the term non-completion or premature termination is used in the current study, rather than therapy dropout.

Findings emphasize the importance of immediate interventions for children with PSBs (1, 7, 8) that actively involve caregivers (1, 9, 10). Short-term cognitive behavioral therapies [ex: CBT- for children with Sexual behavior problems (SBP)] (3) focusing on the child's sexual behaviors have been further validated empirically and appear to be evidence-based practices for children with PSBs (1, 8, 9, 11). In fact, studies focusing on treatment outcomes report a significant reduction in PSBs (5, 8, 9) and in concomitant difficulties (internalized and externalized behaviors, symptoms of dissociation and post-traumatic stress disorder) (5). However, despite the positive treatment outcomes for children with PSBs, therapy non-completion or premature termination remains a recurring problem within clinical populations, significantly hindering our capacity to evaluate their effectiveness, or the opportunity for children to learn to regulate such behaviors and for parents to reinforce the use of appropriate coping strategies (12–15).

High attrition rates, ranging between 30% and 50% of study samples are observed in children and youth dealing with a wide range of problems and receiving different types of treatment [ex: TF-CBT, parent-child interaction therapy (PCIT) (16–18)]. Studies on therapeutic interventions aimed at children with PSBs also show high non-completion rates, ranging from 19% to 37% of samples (3–6) suggesting they face similar challenges regarding the retention of their participants. In fact, previous studies focusing on PSBs have dealt with smaller sample sizes due to significant loss of participants during follow-up (3, 4, 19, 20) that may result from instability within families with children having PSBs (21). Studies evaluating treatments for children with PSBs (3, 4) did not find sociodemographic or distress differences between individuals who completed the treatment and those who did not. However, no studies to our knowledge have specifically focused on identifying predictors of therapy completion for children with PSBs. Therefore, considering the scarcity of therapy completion data focusing on children with PSBs, studies documenting attrition rates in related populations were documented, including children and youth with various difficulties such as post-traumatic stress and externalized or internalized behaviors (15, 16, 18, 22–26), as well as teenage victims of sexual assault (12). Fernandez and Eyberg (25) identified that low socio-economic status, maternal negative talk, less maternal praise pre-treatment and maternal distress were related to attrition. Implementing PCIT, Webb et al. (18) related attrition to parent's readiness to change and level of motivation. Similarly, Abella and Manzano (27) observed that parent's motivation towards the therapy was associated with completion. Differences between completers and non-completers also emerge from other studies. The caregiver and the child younger age (15, 23), as well as the externalized behaviors of the latter would be associated with higher non-completion rates (12, 14, 23). Children with lower self-esteem are also more likely not to complete therapy (13). Furthermore, caregiver stress was linked to non-completion (15, 26) and to the treatment credibility being rated as low by both the child and the caregiver (15). The results from a study conducted by Sprang and colleagues (14) suggest that ethnicity influenced treatment completion. Therefore, they propose that targeted services addressing cultural differences could be helpful at reducing possible obstacles to treatment completion, thus increasing family's engagement (14). In contrast, foster care placement was associated with completion of treatment (6, 14, 23, 24). According to Danko and colleagues (22), children who completed therapy came from more educated families and two-parent households. However, other findings suggest that no individual characteristic of the children, caregivers and families appear sufficient to lead to therapy non-completion, suggesting that an accumulation of several risk factors would best explain greater propensity towards non-completion (28, 29). Altogether, literature reviews show that a large variety of contextual (ex: low SES, number of child trauma, child protection involvement, placement), familial (ex: parent's motivation, maternal talk, distress) and personal factors (ex: age, low self-esteem symptoms) have been explored in relation to therapy non-completion, demonstrating variable results, supporting the hypothesis of cumulative risk factors. For children with PSBs, although studies have not explored factors associated to attrition, research shows that they often manifest concomitant difficulties that may be a representation of such cumulative risk factors, namely a history of victimization in a large proportion, including sexual assault (5, 30, 31) and externalized, and internalized behaviors as well as symptoms of post-traumatic stress (30, 32).

### 1.1 Aim of the study

This study aims to explore whether factors that have been related to therapy non-completion in past research and other populations, also influence non completion of therapy for families with children with PSBs. As high attrition rates have been reported in previous interventions that are similar to the ones implemented for children with PSBs, this study inquires to identify risk factors that make families more likely or not, to complete therapy. More specifically the present study had two objectives. First, at the bivariate level, the objective was to identify individual (gravity and severity of PSBs, behaviors, symptoms, coping mechanisms), familial (support offered, income, caregiver psychological distress) and contextual factors (ex: living situation) that can distinguish between children who complete therapy and those who do not complete therapy. Second, at the multivariate level, thus while considering multiple factors simultaneously, the objective of the study was to identify factors predicting therapy completion.

## 2 Method

### 2.1 Sample

The sample is composed of 67 caregiver-child dyads referred to specialized services for PSBs offered in a metropolitan area of Montreal, Canada. The children were aged 12 and under when the request for services was made and had to be accompanied by a caregiver. Some children (*n* = 5) had reached 13 years old by the time they began therapy. The sample is composed of 23 girls and 44 boys.

### 2.2 Procedure

The 67 caregiver-child dyads were recruited in a specialized center offering therapy to children exhibiting PSBs. Both caregivers and children completed a series of questionnaires during individual interviews carried out with a research assistant and a clinician before starting therapy. These questionnaires were completed by the entire sample. The therapy center in which participants were assessed, offer individual therapy to children who are victims of child sexual abuse using Trauma focused cognitive behavioral therapy [TF-CBT; (33)]. For children with PSB a manualized cognitive behavioral therapy for children with Sexual behavior problems (CBT-SBP) was used (3); with a trauma-focused component when necessary. This TF-CBT approach was initially discussed by Cohen et al. (34) for children with behavior problems and more recently by Allen (35) for children with PSB. Very similar in terms of objectives and therapy components both CBT-SBP and TF-CBT-SBP actively involves caregivers. Treatment fidelity was only partly ensured by use of a manualized guide for therapist, establishing the objectives of each therapy sessions, while providing clinical tools and exercises; and by requesting clinicians to fill out an implementation form regarding the components of therapy. On average, the therapy sessions of our participants consisted of 12 weekly sessions lasting 60 min each. The main goals of the therapy are to reduce PSBs by focusing on risk and maintenance factors and increase the child's self-regulatory abilities. This research project was approved by the responsible ethics committees of University of Sherbrooke and of University of Montreal.

### 2.3 Measures

#### 2.3.1 General information

General information regarding the family's sociodemographics and the child's living situation was retrieved from a questionnaire completed by the caregiver. Missing data for 13 participants regarding family income was replaced by the mean to optimize the number of participants. This strategy helps to avoid deletion of cases when the sample size is already limited, as well as to maintain statistical power [e.g., (36)].

#### 2.3.2 Variety and contextual characteristics of PSBs

The child's sexual behaviors (problematic or not) are described using a questionnaire composed of a list of items from the Child Sexual Behavior Checklist [CSBCL; (37)], adapted by Tourigny & Gagnon (38). This measurement tool is filled out by the professional based on the information gathered during the clinical interview with the parent. Sixty-three items are used to assess the presence or absence of normal and problematic sexual behaviors in the child (0 vs. 1). This first section aims to document the diversity of observed sexual behaviors (e.g., excessive interest in sexuality, showing genitalia to adults/children). The tool also describes the contextual characteristics in which these behaviors occur, using 24 items, providing information on the severity of sexual behaviors (e.g., engaging in sexual behaviors with older or younger children; the child's sexual behaviors becoming more frequent, concerning, and intrusive over time). This modified version of the CSBCL uses a three-point Likert scale instead of five, where each item can be answered with “yes”, “no”, or “don't know”. No clinical threshold is established. A Cronbach's alpha coefficient of 0.81 was calculated for the scale assessing the diversity of sexual behaviors, and a coefficient of 0.92 for the scale regarding the severity of these behaviors.

#### 2.3.3 Externalized and internalized behavior problems

Information related to social skills and externalized and internalized behavior problems was collected by using the Child Behavior Checklist [CBCL; (39)]. Comprised of 113 items regarding the child's behavior over the past two months, parent's responses are rated on a three-point Likert-type scale (i.e., 0 = not true; 1 = somewhat or sometimes true; 2 = very true or often true). Within our sample of children with PSBs, we obtained a Cronbach's alpha coefficient of 0.92 for the total score of the scale, which testifies a good internal consistency.

#### 2.3.4 Symptoms of dissociation

To examine symptoms of dissociation among children, the *Child Dissociative Checklist* [CDC V 3.0; (40)] was administered to caregivers. This instrument includes 20 items whose responses are rated on a three-point Likert-type scale (0 = not true; 1 = somewhat or sometimes true; 2 = very true or often true) depending on the child's behavior for the last 12 months. The total score was used in the analyses, indicating the child's global level of dissociation.

#### 2.3.5 Parent's psychological distress

To document the caregiver's psychological distress, a short version of *L'indice de détresse psychologique de l'enquête Santé-Québec* (41) was used. This instrument provides a measurement of parent's levels of depression, anxiety, irritability, and cognitive problems; the total score thus leading to a global measure of psychological distress. This instrument consists of a 14-items questionnaire that can be answered on a four-point Likert-type scale ranging from 1 (“never”) to 4 (“very often”). Each item must be rated based solely on the previous week. Psychometric qualities have been validated with adult and adolescent populations (42). A very satisfactory Cronbach's alpha of 0.91 was obtained for the sample.

#### 2.3.6 Anxiety

To collect information regarding anxiety, the *Revised Children's Manifest Anxiety Scale* [RCMAS; (43); in French translated version of Hébert & Parent (44)] was administered to children. This version includes 11 items referring to statements to which the child indicates if it is true or false, resulting in a total score ranging from 0 to 11 that allows to determine the level of anxiety experienced by the child.

#### 2.3.7 Depression symptoms

Regarding depression symptoms, the *Children's Depression Inventory* [CDI; (45); French translated version of St-Laurent (46)] was administered to the children. This version includes 10 items, each represented by three different statements from which the child must choose the most representative [e.g., (a) “I am sad sometimes”; (b) “I am sad most of the time”; or (c) “I am always sad”]. The items concern various themes (such as sadness, fears, hope, etc.) that allow to determine a total score regarding depression.

#### 2.3.8 Coping style

The short version of the *Self-Report Coping Style* [SRCS-S; (47); in French translated version of (48)] was administered to children. This revised questionnaire includes 21 items regarding coping strategies used based on a stress factor. Each item can be answered on a five-point Likert-type scale ranging from 0 (“never”) to 5 (“always”). The scale allows to define coping more generally, either as avoidance or approach coping. In the present study, we also used more specific coping strategies within each of these two subscales, Distinancing being a form of avoidance coping and search for social support a forn of approach coping strategy. In this case Distancing coping refers to items such as (e.g., I pretend it never happened; I tell myself it doesn't bother me). This measure has good psychometric properties (48).

#### 2.3.9 Maternal and general support

To explore maternal and general support, the *Children's Impact of Traumatic Events Scale* [CITES II; (49)] was administered to children. This instrument consists of a 55-items questionnaire that can be answered on a three-point Likert-type scale (0 = not true; 1 = somewhat true; 2 = very true). The answers are based on a trauma previously identified with the child and scores are therefore available for a smaller number of children.

#### 2.3.10 Self-Esteem

The French version revised by Hébert and Parent (50) of the *Self-Perception Profile For Children* [SPPC; (51)] was used to assess the child's self-esteem level. The 6 items consist of two affirmations; the child must first choose which affirmation represent them more (51) and secondly, whether this description is a “bit like them” or “a lot like them”. This measurement instrument is widely used by experts and demonstrates good internal consistency (52, 53). Cronbach's alpha for this sample of children with PSBs is 0.58.

#### 2.3.11 Implementation variables

Number of therapy sessions completed; completion/non-completion of therapy and motives of premature completion of therapy were documented via an implementation questionnaire filled out by the therapist in charge. All non-completers were recoded in the non-completion category regardless of motive.

### 2.4 Analytical strategy

The analytical strategy unfolds in two sections. First, bivariate analyses are conducted to compare children who completed the therapy with children who did not regarding various factors, namely sociodemographic characteristics (i.e., child's age and sex; the family's annual outcome), the nature of the PSBs (i.e., variety and contextual characteristics), the child's individual characteristics at the beginning of therapy (i.e., internalized and/or externalized behaviors; self-esteem; anxiety and depressive symptoms; relational difficulties; level of dissociation symptoms and support and coping mechanisms), as well as the parent's psychological distress. The selection of these variables follows a conceptual reasoning that reflects knowledge from the empirical literature. For these bivariate analyses, *t*-tests were conducted with the continuous variables, while chi-squared tests were executed with the categorical variables. Second, multivariate analyses are conducted (i.e., binary logistic regression models) to identify factors associated to therapy completion. Forced entry was chosen considering the small sample size to avoid overfitting of the model to our data [e.g., (54)]. The variables entered in regression models were based on results of the bivariate analyses (e.g., *t*-test, chi-square, correlations), as well as univariate examination of each variables (e.g., to verify for problems in terms of insufficient sample size or zero cell). Given the constraints of our sample size (*n* = 67), which allows for the inclusion of approximately six variables in the multivariate analysis, we prioritized variables that demonstrated significant associations in the bivariate analyses. These variables included Coping Distancing, Coping Social Support, Out-of-home Placement, and Maternal Support. However, Maternal Support was excluded due to the presence of missing data. To ensure a well-specified model and reduce potential biases, we also included Family Income and Age, based on their relevance in the literature. These variables help control for potential confounding and interaction effects that may not be captured by the other predictors. Furthermore, considering the conceptual framework and the nature of the outcome variable, it was essential to include a variable representing the therapy motive—specifically, the level of PSB variety—as this factor is theoretically linked to therapy completion. Including this variable allows us to account for the variability in therapy outcomes based on the diversity of PSB presented by the participants. The results were considered based on the odds ratio (OR), which allow to examine to what extent a factor significantly increases (OR > 1) or decreases (OR < 1) the likelihood that the outcome of interest occurs (55), that is the therapy completion.

## 3 Results

### 3.1 Sample characteristic and comparisons based on therapy completion and non-completion

Table 1 presents the distribution of the scores for the entire sample (first column), along with the results of the chi-square tests. At the time of assessment, 46.3% of children were living in an out-of-home placement. More than one child in five came from a single-parent family, 16.4% from a blended family, and more than a third lived in a low-income household. For close to a third of the sample, the mother was employed, while the father was in less than half of the cases. The accompanying adult was a biological parent in more than half of the sample, and most of them were female (83.6%). Regarding the child's individual characteristics, descriptive analyses indicated that just under a third of the sample obtained a clinical score for relational difficulty (31.3%), and dissociation (32.8%). After thorough assessment, a non-completion of therapy rate of 31.4% reduced the sample for completed therapy to 46 dyads. Children completed between 0 and 25 therapy sessions, with an average of 11 sessions (SD = 4.79), while the median stands at 12 sessions. Twenty-one children did not complete therapy, of which 12 (57.1%) the reasons reported by clinicians were categorized as dropout. The underlying reasons for withdrawal of therapy for the remaining 43% remain undefined. Descriptive results also indicate that 64.6% of the children in the sample presented externalized behaviors (e.g., aggressive, delinquent behaviors) in an amount that reached the clinical level measured before therapy. This percentage of clinical level scores is much higher than any other form of psychological distress (49.3 presented internalized behaviors like anxiety, depression, etc.) which were closer to 20%.

**Table 1:** Bivariate comparisons with crosstables comparing therapy completers and non-completers (*n* = 67).

Variables	Therapy outcomes			*X^2^*	ϕ
	Total sample 67 (100%) *N* (%)	Non-completion 21 (31%) *N* (%)	Completion 46 (69%) N (%)		
Sex (*n* = 67)				1.81	0.13
Female	23 (34%)	5 (23.8)	18 (39.1)		
Male	44 (66%)	16 (76.2)	28 (60.9)		
Living situation (*n* = 67)				3.85	**0** **.** **24** *
Biological family	36 (54%)	15 (71.4)	21 (45.7)		
Out-of-home placement	31 (46%)	6 (28.6)	25 (54.3)		
Mother's occupation (*n* = 67)				0.11	0.04
Employed	21 (31%)	6 (28.6)	15 (32.6)		
Non employed	46 (69%)	15 (71,4)	31 (67.5)		
Mother's level of education (*n* = 58)				0.00	−0.01
Highschool diploma or lower	31 (53%)	10 (52.6)	21 (53.8)		
Post-high school diploma	27 (47%)	9 (47.4)	18 (46.2)		
Father's occupation (*n* = 67)				1.08	0.13
Employed	32 (48%)	9 (42.9)	26 (56.5)		
Unemployed	35 (52%)	12 (57.1)	20 (43.5)		
Father's level of education (*n* = 47)				0.74	0.13
Highschool diploma or lower	26 (55%)	8 (47.1)	18 (60.0)		
Post-high school diploma	21 (45%)	9 (52.9)	12 (40.0)		
Family's annual income (*n* = 67)				1.58	−1.17
29,999$ and less	38 (56%)	15 (75.0)	30 (88.2)		
30,000$ and more	29 (43%)	5 (25.0)	4 (11.8)		
Child's clinical level symptoms					
Internalized behaviors clinical level^a^	33 (49,3)	8 (38.1)	25 (54.3)	1.52	.151
Externalized behaviors clinical level^a^	43 (64,6)	13 (30.2)	30 (69.8)	.069	.032
Relational difficulty clinical level^a^	21 (31.3)	5 (23.8)	16 (34.8)	.807	.110
Dissociation symptoms^a^	22 (32.8)	7 (33.3)	15 (32.6)	.003	−.007
Anxiety symptoms^a^	12 (17.9)	4 (19.0)	8 (17.4)	.027	−.020
Depression symptoms^a^	8 (11.9)	1 (14.8)	7 (15.2)	1.5	.15

Bold text indicates significant variables.

^a^ These variables refer to the proportion of children who obtained scores on related-assessment tools that indicated a clinical level.

* *p* < .05.

Bivariate comparisons (see Table 1, column 2 and 3) between children who completed the therapy and those who did not were conducted to identify characteristics that distinguish the two groups. There were no significant differences between completers and non-completers in terms of sex, parent's level of education or occupation and familial income. Results indicate that a higher percentage of children who completed therapy live out of home, in a placement situation (54.3%) when compared to those who live with their biological parents (45.7% of completers).

Bivariate comparisons between children who completed the therapy and those who did not are also presented in Table 2, for continuous variables. Based on the results from the bivariate analyses, while as a general rule, completers presented slightly lower mean rank scores of psychological distress on all measures (internalized and externalized behaviors, depression, anxiety, relational difficulties, dissociation and accompanying parent psychological distress), as well slightly higher self-esteem ratings. These perceived differences between groups were however not statistically significant within our sample for the majority of variables, with the exception of the reported use of coping strategies (distancing and social support) by children and the perceived maternal support.

**Table 2:** Bivariate comparisons with *t*-test comparing characteristics measured before therapy between completers and non-completers (*n* = 67).

Variables	Total Sample *N* = 67	Non-completion(*n* = 21) M(*s.-d.)*/	Completion (*n* = 46) M(*s.-d.)*/	*t*	F	df
Sociodemographics						
Age	9.16 (2.21)	9.48 (2.14)	9.02 (2.25)	.413	.41	65
Family's income (1–8)	4.13 (2.20)	4.09 (2.40)	3.82 (1.84)	.503	1.96	66
Nature of problematic sexual behaviors						
Variety (A) (0–59)	15.83 (10.47)	16.22 (11.35)	15.64 (10.16)	.209	.290	64
Contextual characteristics (B) (0–24)	7.22 (5.50)	5.95 (4.84)	6.72 (5.21)	−.553	.256	63
Child's individual characteristics						
Internalized behaviors	61.94 (9.71)	60.76 (8.29)	62.48 (10.35)	−.66	3.84	65
Externalized behaviors	66.60 (9.85)	65.76 (10.65)	66.98 (9.55)	−.46	.76	65
Relational difficultiess		64.24 (8.15)	65.22 (11.08)	−.36	3.53	65
Self-esteem	19 (3.61)	19.00	19.04	−.045	1.95	65
Anxiety symptoms	4.9 (2.91)	4.67 (3.25)	5.02 (2.79)	−.459	.80	65
Depression symptoms	3.25 (3.01)	2.76 (2.14)	3.48 (3.33)	−.90	1.66	65
Dissociation	9.16 (5.87)	8.24 (5.75)	9.59 (5.95)	−.87	.009	65
Coping avoidance	9.42 (2.70)	9.95 (2.42)	9.17 (2.82)	1.05	.98	65
Coping approach	11.99 (3.82)	11.10 (3.56)	12.39 (3.90)	−1.29	.301	65
Coping social support	10.19 (4.92)	8.52 (4.93)	10.96 (4.77)	**−1** **.** **92** *	.385	65
Coping distancing	10.21 (3.67)	11.81 (3.02)	9.48 (3.74)	**2** **.** **50** *	1.67	65
Parent's difficulty						
Psychological distress	23.77 (20.03)	22.02 (17.7)	24.79 (21.47)	-.487	0.36	65
Child's support following disclosure						
Maternal support	4.97 (1.41)	4.15 (2.27)	5.50 (1.24)	**−1** **.** **96** *	7.65	31
General support	3.91 (1.66)	3.46 (1.33)	4.19 (1.81)	−1.35	2.69	32

Bold text indicates significant variables.

* *p* < .05.

### 3.2 Therapy completion and associated factors

Guided by results from the bivariate analyses^1^, a logistic regression model was performed to examine the relationship between coping mechanims and the child's living situation (i.e., out-of-home placement) as independent variables; and therapy completion as the outcome variable. The analysis was conducted while controlling for sociodemographics (i.e., child's age and family's annual outcome) and the nature of the child's PSBs (i.e., variety of PSBs^2^) to account for potential confounding effects (see Table 3). The model was found to be statistically significant (χ^2^ = 15.14, *p* < .019). The odds ratio for distancing coping was (.80), indicating a significant [negative] association with the likelihood of therapy completion, indicating that therapy completion is less likely as use of distancing increases. Results also indicate that the odds ratio for living in a placement situation was (4.32), indicating a positive association with the likelihood of therapy completion. In other words, placement increases the odds of therapy completion by 4.3 times. Altogether, the model's Nagelgerke's pseudo R^2^ was moderate, explaining 28% of the variance in treatment completion. The non-significant Hosmer-Lemeshow test (1.86) confirms that the differences between observable modalities and those predicted by the model adequately fit the model. The model is efficient to properly classify 72% of the population.

**Table 3:** Logistic regression model for therapy completion (*n* = 66).

Indicators	B	SE	Wald	*p*	Exp (B)	95% C.I.
Sociodemographics						
Family's annual income^a^	−.274	.178	2.38	.12	.76	.54–1.08
Age^a^	−.214	.155	1.90	.17	.81	.60–1.09
Child's behavior						
PSBs Variety (section A)^a^	−.055	.035	2.42	.12	.94	.88–1.01
Coping Distancing	−.212	.102	4.27	.039	**.** **81** *	.66–.99
Coping social support	.070	.069	1.04	.31	1.07	.94–1.23
Child's living situation						
Out-of-home placement	1,46	.696	4.43	.035	**4** **.** **33** *	1.11–16.96
Constant	5.71	2.65	4.64	.031	304.32	
Model fit						
Hosmer-Lemeshow goodness-of-fit	1.87					
Chi-Square	15.14*					
Classification	72.2%					
Nagelkerke R^2^	.287					

Non completers = 0 (*n* = 21), Completers = 1 (*n* = 45).

Bold text indicates significant variables.

^a^ These indicators were added in the model as control variables.

* *p* < .05.

## 4 Discussion

Therapy completion is a challenge for therapeutic programs offered to children experiencing various difficulties (3, 4, 12, 15, 17, 22, 23, 28, 56). In the current study, therapy completion for children with PSBs is no exception, with a 31.4% rate of non-completion. This considerable loss of participation could reflect the instability experienced by families with a child with PSBs (21). As the scientific literature points out (1, 5, 21, 30, 31), these families often experience several situations of adversity, which could potentially impact their mobilization for therapy. This research first sought to identify if children with PSB who did not complete psychotherapy differed from those who completed it, regarding personal and contextual factors. The second objective was to explore personal and contextual factors associated with completion while considering multiple factors simultaneously and controlling for PSB variety and family income.

When comparing completers to non-completers, while the groups did not differ in terms of socio-demographic characteristics (e.g., gender, income), in terms of the child's mental health (e.g., depression, anxiety, dissociation) and externalized behaviors or the nature of PSBs; they differed in terms of child protection involvement (placement) and in terms of perceived maternal support and coping mechanisms, with non-completers describing more distancing and reaching less often for social support. When considering all these factors together, while controlling for age, income and the characteristics of PSB's; placement by child protection services and less use of distancing coping came out as the two most important factors associated to completion. Unfortunately, maternal support had to be excluded from these analyses because of missing data, considering that many children are in a placement situation.

The results reveal that being in a family situation of placement during psychotherapy sessions is associated with a higher rate of completion. This finding is in line with those of previous studies (7, 14, 24) suggesting that children in out of home placement may benefit from external monitoring and support, such as transportation to sessions (7) which contribute to increasing treatment attendance. This could be partially explained by the fact that PSBs are often manifested alongside several adjustment difficulties (5, 30, 32, 57) which might imply that their parents are very solicited and therefore decrease their mobilization in therapy. Children with PSBs also cumulate adverse family life experiences (e.g., financial difficulties, substance abuse, domestic violence) (1, 5, 21, 30, 31) that can impact parent's ability to engage in therapy.

Aside from living situation, results revealed that while making less use of distancing coping is associated with therapy completion, non-completers tend to depend less on social support than completers. They also report feeling less maternal support when confronted with a difficult experience. These results are likely to reflect elements that may have prevented early termination, had they been available; and suggest that a motivational approach (e.g., exploring goals, exploring pros and cons for change, identifying motivations for therapy) may be beneficial to both children and their parents.

Similarly to previous studies on children with PSBs (3, 4), the current findings indicate that completers and non-completers are very similar in terms of personal, familial and contextual factors. The fact that no difference emerged regarding the nature of PSBs (variety and severity) and the caregivers' psychological distress differs from a study conducted by Nathanson and colleagues (26) reporting that parent stress was one of the most commonly reasons for not completing therapy. However, parent stress was cited by parents themselves to explain early termination (26), which was not done in the current study.

While the present study did not identify differences between completers and non-completers regarding externalized behaviors, the presence of such difficulties has been associated with therapy non-completion in a past studies (12, 14, 23). This may be explained by low variance on this factor, as all of the children from this sample presented high levels of externalized behaviors. The absence of significant difference regarding symptomatology (anxiety, depression, dissociation), however, has been replicated in other studies (24, 26, 58) which suggest that there is no evidence concerning a possible effect of this variable on therapy completion. In line with these findings, the terminology non-completion seems to be indicated considering that the child's characteristic do not allow to explain why they did not complete therapy and the possible impact that external factors may play in their session's attendance. To further explore this hypothesis, it would be beneficial to gather additional data on the therapy implementation, such as the reasons for not coming to sessions as well as feedback during and after therapy. Nathanson and colleagues (26) suggested to add a measure to document the child and caregiver experience to allow both the completers and non-completers to share what they did or did not appreciate about the intervention. Better understanding their experience could therefore allow for a more individualized intervention that target their specific needs and reduce the possible obstacles they may encounter in attending therapy sessions.

The scientific literature highlighted some variables predicting therapy non-completion, such as sociodemographic variables (13, 22, 23) and externalized behaviors (12, 23). This study does not find the same results as previous research. While the influence of child protection involvement has been infrequently studied, our findings highlight the importance of organization and structure provided by these services in facilitating therapy completion. Living in a placement situation predicted successful therapy completion, suggesting that children in such environments benefit from the structured support of child protection and the proactive involvement of foster care parents. Additionally, child protection services and placements may encourage children to engage in more problem-solving behaviors and reduce avoidance strategies. These results are in line with the argument that the accumulation of risk factors, which may be less present in families offering placement, would lead to a greater propensity of not completing therapy (28, 29).

In line with our findings, it would appear relevant in subsequent studies to focus on a variety of factors that may also influence therapy completion, including the biological family context and the opinions of the child or the parent. Future studies could explore in more details what families feel and how that influences therapy completion. While the current study explored family and parental factors, the available variables may have been limited to identify the challenges that parents experience. What obstacles do they encounter? What could be done in a concrete way so that these families are more involved in psychotherapy? Addressing upfront what may be an obstacle for families to come to psychotherapy and trying to identify potential solutions should be an avenue to consider for practice (14, 28). Thus, working in concert with the people who make it possible to daily implement the key components of the interventions is critical for the psychotherapy to be effective (1, 14, 20). Ensuring that caregivers are psychologically available (21), actively involved and motivated to help their child through the whole process should guide the efforts in terms of intervention for children with PSBs. Moreover, given the central role that therapeutic alliance plays in treatment outcomes (24, 59) it would be pertinent in future research to assess the possible effect it might have on participants retention.

### 4.1 Study's strengths and limitations

The results of our study may have been influenced by the sample size (*n* = 67). Aside from limiting the number of factors that could be analysed in the multivariate model, this limitation could have reduced the statistical power of the analysis and prevent the detection of differences between individuals. Further, from the outset, it should be noted that research focusing of therapy completion face a methodological challenge stemming from the lack of an operational definition of dropout (15, 27). Therefore, more specific criteria may help in developing a more systematic and operationalized definition of non-completion, while documenting and accounting for motives of withdrawal or dropout. In fact, the available data didn't precisely document the reasons as to why the participants did not complete therapy, thus making it possible that distinct profiles were joined in the same category despite their differences. In that regard, having access to qualitative data could be interesting in future studies to better understand the reasons for not completing therapy. Even though the variables used for the analysis were chosen considering past studies results, it was not possible to document the possible effect of certain variables that were found to be associated with therapy completion, such as therapeutic alliance (59). Finally, given the sample size that reduces analysis statistical power and the results generalizability, more studies should be conducted to better understand why an important proportion of children with PSBs do not complete therapy.

## 5 Conclusion

In our sample of children with PSBs, all together, our results indicate that children who live out-of-home in a placement situation and who use less distancing coping are more likely to complete their therapy than those who live with their biological parents. Further, compared to non-completers, those who complete therapy appear to rely more on social support. Therefore, these aspects deserve close attention early in the therapeutic process to prevent premature termination. Considering the current findings, in addition to consider children's characteristics, it might be beneficial to focus more extensively on factors that are external to the child in future studies, such as factors influencing the family as a unit and the caregivers' implication and mobilization for therapy (14, 17, 26). Unfortunately, as challenges appear to add up for families, therapy retention is at risk (17, 28). This aspect could probably be considered and addressed more closely with families beginning a therapeutic program. Future research could further explore treatment retention strategies implemented by treatment centers to better anticipate such risk. In this sense, the quality of the therapeutic alliance with both parents and children could be explored (14, 24). Let's hope that knowledge documenting how to better support children with PSBs and their families will continue to expand and will translate into advances in practice that will allow each child to benefit from targeted interventions.

## Data Availability

The original contributions presented in the study are included in the article/Supplementary Materials, further inquiries can be directed to the corresponding author.

## Footnotes

^1^While the variable referring to the maternal support was significant in the bivariate analyses, this variable was not added to the regression model because of a problem in terms of insufficient sample size.

^2^The PSBs variety and contextual characteristics were identified as being correlated. Therefore, only the variety score was added to the regression model.
